# Epidemiology of dengue reported in the World Health Organization’s Western Pacific Region, 2013–2019

**DOI:** 10.5365/wpsar.2023.14.1.973

**Published:** 2023-03-22

**Authors:** Eri Togami, May Chiew, Christopher Lowbridge, Viema Biaukula, Leila Bell, Aya Yajima, Anthony Eshofonie, Dina Saulo, Do Thi Hong Hien, Satoko Otsu, Tran Cong Dai, Mya Sapal Ngon, Chin-Kei Lee, Reiko Tsuyuoka, Luciano Tuseo, Asheena Khalakdina, Vannda Kab, Rabindra Romauld Abeyasinghe, Rajendra Prasad Yadav, Princess Esguerra, Sean Casey, Chun Paul Soo, Munehisa Fukusumi, Tamano Matsui, Babatunde Olowokure

**Affiliations:** a Health Emergencies Programme, World Health Organization Regional Office for the Western Pacific, Manila, the Philippines.; b World Health Organization Representative Office for Lao People’s Democratic Republic, Vientiane, Lao People’s Democratic Republic.; c Division of Programmes for Disease Control, World Health Organization Regional Office for the Western Pacific, Manila, the Philippines.; d World Health Organization Representative Office for Viet Nam, Hanoi, Viet Nam.; e World Health Organization Representative Office for China, Beijing, China.; f World Health Organization Representative Office for Cambodia, Phnom Penh, Cambodia.; g World Health Organization Representative Office for the Philippines, Manila, the Philippines.; h Division of Pacific Technical Support, World Health Organization, Suva, Fiji.; i World Health Organization Representative Office for Malaysia, Cyberjaya, Malaysia.

## Abstract

The global burden of dengue, an emerging and re-emerging mosquito-borne disease, increased during the 20-year period ending in 2019, with approximately 70% of cases estimated to have been in Asia. This report describes the epidemiology of dengue in the World Health Organization’s Western Pacific Region during 2013–2019 using regional surveillance data reported from indicator-based surveillance systems from countries and areas in the Region, supplemented by publicly available dengue outbreak situation reports. The total reported annual number of dengue cases in the Region increased from 430 023 in 2013 to 1 050 285 in 2019, surpassing 1 million cases for the first time in 2019. The reported case-fatality ratio ranged from 0.19% (724/376 972 in 2014 and 2030/1 050 285 in 2019) to 0.30% (1380/458 843 in 2016). The introduction or reintroduction of serotypes to specific areas caused several outbreaks and rare occurrences of local transmission in places where dengue was not previously reported. This report reinforces the increased importance of dengue surveillance systems in monitoring dengue across the Region.

The global burden of dengue, an emerging and re-emerging mosquito-borne disease, increased from 2000 to 2019. (*1*) An estimated 70% of dengue virus infections are thought to occur in Asia. (*2*) It has previously been reported that in the World Health Organization’s (WHO’s) Western Pacific Region, the number of dengue cases increased from approximately 200 000 in 2008 to more than 450 000 in 2015. (*3*) During this period, several countries and areas in the Region experienced large-scale outbreaks. (*4*-*6*)

Dengue is a public health threat because it is associated with large outbreaks in communities, severe disease and mortality. (*1*) Host immunity factors, such as serotype interaction, antibody-dependent enhancement and cross-immunity, complicate the clinical course, which leads to challenges in managing severe cases. (*1*, *7*) Additionally, socioeconomic and environmental factors, including climate change, drive disease transmission and complicate prevention and control activities.

In response to these challenges, a revised Western Pacific Regional Action Plan for Dengue Prevention and Control was developed and endorsed at the 67th meeting of the Regional Committee for the Western Pacific in October 2016. (*3*) The Plan has guided countries and areas in the Region on improving the laboratory diagnosis of dengue, and the clinical management, surveillance and sustainable vector management for the disease to reduce morbidity and mortality, and decrease impacts on health systems.

Sharing information and data about dengue helps countries and areas better understand transmission patterns and supports the implementation of dengue prevention and control measures. (*2*) As a continuation of previous Regional dengue epidemiology updates in 2010, 2011 and 2012, (*8*-*10*) this analysis reports data collated by the WHO Regional Office for the Western Pacific to describe the epidemiology of dengue in the Region from 2013 to 2019 using regional surveillance data. Data from 2020 to 2021 were excluded due to changes in reporting practices, population movement and people’s behaviours as a result of the COVID-19 pandemic.

## Methods

Regional dengue data from 2013 to 2019 were collated from indicator-based surveillance systems from countries and areas in the Region. Information was also collected about laboratory sampling schemes and the confirmation methods used by each country and area. Data were either sent to WHO by ministries of health or collected from official web sites where they were publicly available. Additional data – including serotype information, case definitions, and the numbers of clinically confirmed cases, laboratory-confirmed cases and imported cases and deaths – were provided by Australia, Cambodia, Japan, the Republic of Korea, Malaysia, New Zealand, Pacific Island countries and areas (PICs), the Philippines, Singapore and Viet Nam. Information was reported based on the standard dengue case definitions used in each country or area (**Table 1**). Missing data were supplemented by using official dengue outbreak situation reports published on ReliefWeb (https://reliefweb.int/), manuscripts identified through PubMed using keywords [“dengue” AND “outbreak” AND “(country/area name)”], yearly aggregated data collected from all countries and areas in the Region through International Health Regulations (2005) channels, and WHO Regional biweekly dengue reports. (*18*)

**Table 1:** Dengue clinical case definitions, and laboratory sampling and testing methods used for surveillance in countries, WHO Western Pacific Region, 2019

Country	Case definition^a^		Laboratory sampling and testing method	Surveillance and case reporting
	Clinically confirmed case	Laboratory confirmation required		
Australia (*11*)	Fever, headache, arthralgia, myalgia, rash, nausea or vomiting	Yes	NS1, IgG seroconversion, IgM detection, nucleic acid or virus isolation. All clinically diagnosed cases have laboratory testing.	All confirmed cases require both laboratory-definitive evidence and clinical evidence. Both confirmed and probable cases are nationally notifiable.
Cambodia (*12*)	Suspected dengue: high fever (39–40 °C) for 2–7 days (usually 3–4 days), with two or more of the following signs: flushed face, headache, retro-orbital pain, myalgia or arthralgia, cutaneous rash, haemorrhagic signs (e.g. petechiae, positive tourniquet test) and leucopoenia Probable dengue: signs of suspected dengue plus laboratory test results (right column) or a case that occurred in an area where a dengue case has been confirmed	No	Data are collected for the Cambodia Laboratory Information System, composed of 32 hospital laboratories where NS1 detection is conducted. Laboratory testing: Antibody haemagglutination inhibition ≥ 1/1280 or IgM- or IgG-positive by ELISA in convalescent serum.	Suspected cases are reported from all national hospitals and all provincial hospitals, but not from private clinics.
China^b^	More than two of the following symptoms: acute onset fever, severe headache, orbital pain, myalgia, arthralgia, fatigue, a history of travel in a dengue-endemic area during the 15 days before symptom onset or cohabitation with an individual with confirmed dengue, or no travel history but with a rash or positive tourniquet test AND leucopoenia or thrombocytopenia or serum IgM positivity	No	Real-time PCR, NS1 in acute-phase serum or virus isolation from an acutely infected patient’s serum.	Both clinically confirmed and laboratory-confirmed cases are notified as an infectious disease.
Japan (*13*)	Symptoms including acute onset of fever lasting for 2–7 days (commonly biphasic), headache, retro-orbital pain, arthralgia, myalgia, fatigue, conjunctivitis or rash AND laboratory confirmation (right column)	Yes	All clinically diagnosed cases have laboratory testing. Laboratory confirmation requires at least one of the following: a positive PCR test, NS1 detection, serology (IgM, seroconversion) and/or virus isolation.	All reported cases have laboratory testing.
Lao People's Democratic Republic (*14*)	WHO 2009 dengue case classification^c^	No	Laboratory testing is conducted by RDT and PCR on a subset of specimens referred to the laboratories. Serotyping is also conducted on a subset of specimens.	Clinically confirmed cases (dengue with and without warning signs and severe dengue cases) are reported.
Malaysia (*15*)	WHO 2009 dengue case classification^c^	Yes	All suspected cases are tested by the rapid combo test for NS1, IgM and IgG; ELISA for the dengue antigen and serology, real-time PCR for detecting viral RNA, or by viral isolation.	All reported cases have laboratory testing.
New Zealand (*16*, *17*)	Acute onset of fever; headache, particularly retro-orbital; myalgia and arthralgia; and a fine rash, which may be itchy and usually begins on the extremities but spares the palms and soles. Other symptoms include weakness, depression, anorexia, abnormal taste, sore throat, coughing, vomiting and abdominal pain.	No	At least one of the following tests is required for laboratory confirmation: viral isolation, dengue virus (DENV) nucleic acid amplification, IgM or IgG seroconversion, a significant increase in antibodies (fourfold or greater) by serological test.	Both clinically confirmed and laboratory-confirmed cases are reported.
Philippines51–53	WHO 2009 dengue case classificationc In addition, suspected cases are those who were previously well but have acute febrile illness for 2–7 days with clinical signs and symptoms of dengue.	No	A subset of suspected cases have laboratory testing. Confirmed dengue is defined as a suspected case with positive viral culture isolation and/or PCR. Probable dengue cases are NS1- or IgM-positive.	Suspected cases are reported.
Republic of Korea54	Acute onset of fever, headache, arthralgia, myalgia, leucopoenia, thrombocytopenia or bleeding AND laboratory confirmation (right column)	Yes	All clinically diagnosed cases have laboratory testing by real-time PCR or ELISA (IgM).	All reported cases have laboratory testing.
Singapore55	A clinical case meets the criteria of fever, headache, backache, myalgia, rash, abdominal discomfort and thrombocytopenia.	Yes	Samples are tested by the laboratory as ordered by the physician. Laboratory confirmation is done by dengue NS1 antigen testing, IgM or PCR.	All reported cases have laboratory testing.
Viet Nam56	Acute onset of fever lasting 2–7 days AND at least two of the following: haemorrhagic manifestation or presentation, headache, loss of appetite, nausea, vomiting, rash, muscle pain, joint pain, orbital pain, lethargy, abdominal pain	No	MAC-ELISA is conducted for at least 7% of clinical cases and virus isolation is conducted for at least 3% of clinical cases. In an outbreak, at least 5–10 suspected cases are tested.	Both clinically confirmed and laboratory-confirmed cases are reported.

ELISA: enzyme-linked immunosorbent assay; IgG: immunoglobulin G; IgM: immunoglobulin M; MAC-ELISA: dengue IgM capture ELISA; NS1: rapid antigen diagnostic test to detect dengue virus non-structural protein; PCR: polymerase chain reaction; RDT: rapid diagnostic test.

^a^ Only the minimum criteria required for fulfilling a clinical definition of dengue are included here; any additional signs and symptoms required for more severe forms are not listed.

^b^ Data sourced from WHO internal communications.

^c^ In the WHO 2009 dengue classification system, a probable case is any case with fever and two or more of the following: nausea, vomiting, rash, aches and pains, positive tourniquet test, leucopoenia or any warning sign. A case with warning signs is defined as a clinically diagnosed case if they have any of the following: abdominal pain or tenderness, persistent vomiting, clinical fluid accumulation, mucosal bleeding, lethargy, restlessness, liver enlargement > 2 cm, or increase in haematocrit concurrent with rapid decrease in platelet count. Severe dengue is defined as severe plasma leakage leading to any of the following: shock, fluid accumulation with respiratory distress OR severe bleeding as evaluated by clinician OR severe organ involvement of the liver (i.e. aspartate amino transferase or alanine amino transferase ≥ 1000 units/L), central nervous system (i.e. impaired consciousness), heart or other organs.

**Table 1** summarizes the dengue surveillance systems, case definitions, laboratory sampling methods and serotype data. It was not possible to compare trends between countries and areas due to the differences in surveillance methods and reporting practices. The crude regional case notification rate per 100 000 population per year was calculated using the number of cases and deaths reported to WHO and standard calculation methods:

Case notification rate per 100 000 population per year = (*c/p*) × 100 000 and

95% confidence interval = (100 000/*p*) (*c* ± 1.96 × √*c*),

where *c* is the total dengue notification case count in a given year and *p* is the population estimate for the Region in a given year. United Nations population estimate data were used for calculations. Population data for the Pitcairn Islands were not included in the United Nations population database. (*19*) Therefore, we used the closest population estimates based on the Pitcairn Islands’ government web site. In this report, an outbreak is defined as the “occurrence of cases of disease in excess of what would normally be expected in a defined community, geographical area or season.” (*20*)

## Results

**Fig. 1:**
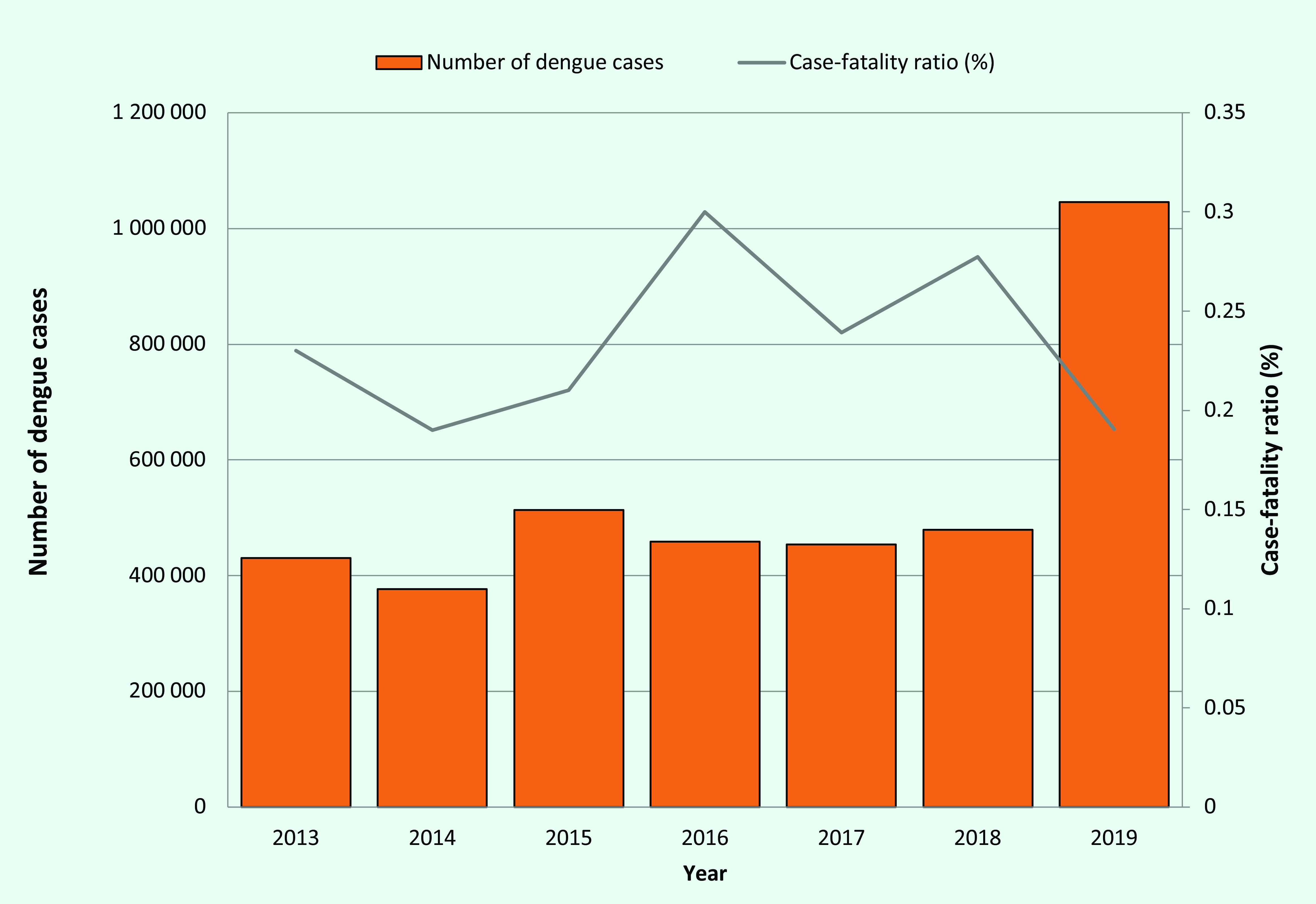
Number of dengue cases and case-fatality ratios reported to WHO from the Western Pacific Region, 2013–2019

**Fig. 2:**
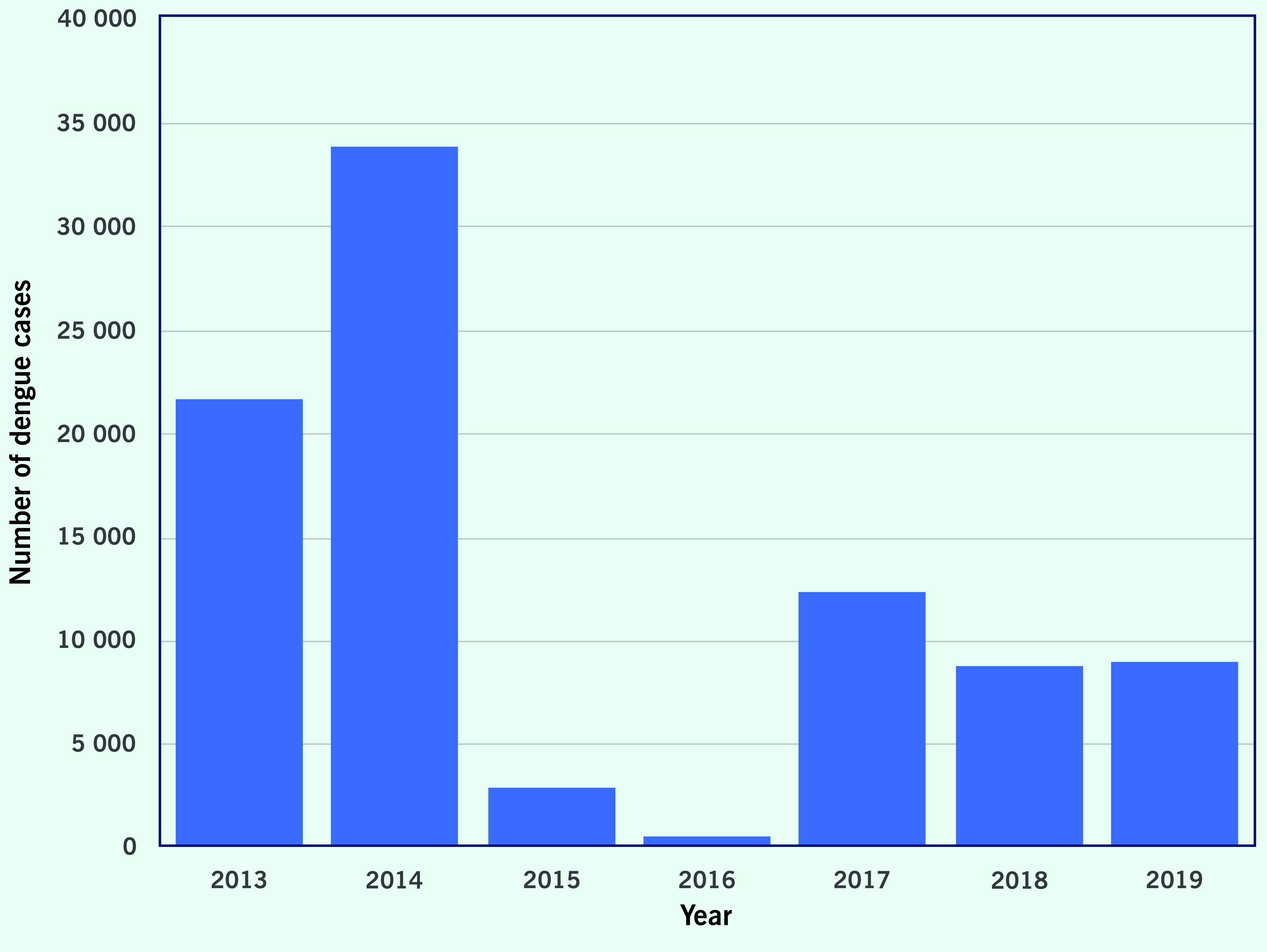
Number of dengue cases reported to WHO from Pacific Island countries and areas, Western Pacific Region, 2013–2019

In the Region, the total number of annual dengue cases reported increased from 430 023 cases from  22 countries and areas in 2013 to 1 050 285 cases from 18 countries and areas in 2019 (data not shown). The lowest annual number of cases during these 7 years was reported in 2014, with 376 972 cases. In 2019, the total number of reported dengue cases surpassed 1 million for the first time. From 2013 to 2019, the case-fatality ratio (CFR) fluctuated between 0.19%  (724/376 972 reported in 2014 and 2030/1 050 285 in 2019) and 0.30% (1380/458 843 reported in 2016) (**Fig. 1**). The number of cases reported from the PICs did not show a clear trend, with more cases reported in 2013 and 2014 compared with 2015 and 2016 (**Fig. 2**). There were challenges in calculating the CFRs for some countries due to limited reporting on dengue cases or deaths associated with dengue, or both.

[**insert** Figure 1]

[**insert** Figure 2]

From 2013 to 2018, the crude annual case notification rates in the Region ranged from a low of  19.82/100 000 population per year (95% confidence interval [CI]: 19.76–19.89) in 2014 to a high of 26.84/100 000 population per year (95% CI: 26.77–26.92) in 2015. In 2019, the case notification rate increased twofold to 53.71/100 000 population per year (95% CI: 53.61–53.81) (**Table 2**).

**Table 2:** Crude regional case notification rates for dengue reported to WHO from the Western Pacific Region,  2013–2019

Year	Number of cases reported in the Region	Population in the Region^a^	Case notification/ 100 000 population per year^b^	95% confidence interval
2013	430 023	1 889 727 401	22.76	22.69–22.82
2014	376 972	1 901 609 413	19.82	19.76–19.89
2015	513 574	1 913 189 733	26.84	26.77–26.92
2016	458 843	1 924 437 124	23.84	23.77–23.91
2017	454 231	1 935 317 876	23.47	23.40–23.54
2018	479 263	1 945 715 729	24.63	24.56–24.70
2019	1 050 285	1 955 495 216	53.71	53.61–53.81

^a^ Population data were extracted from United Nations population estimates. (*19*)

^b^ Crude notification rates in the Region should be interpreted with caution, considering that the risks of disease and population sizes vary substantially across the Region, as well as the surveillance systems used to determine cases of dengue.

From 2013 to 2019, large-scale outbreaks with notable increases in the number of cases were reported in multiple countries. Outbreaks were reported from the PICs every year from 2013 to 2019. There were two notable years, 2017 and 2019, when multiple outbreaks were reported across the Region, including in the PICs, with seven countries reporting outbreaks. All dengue serotypes (DENV-1, DENV-2, DENV-3 and DENV-4) were reported in the Region during the review period. Concurrent infections with two serotypes were reported in some countries. While some countries reported the same predominant serotype from 2016 to 2018, others reported changes in the predominant serotype. Additionally, there were reports of the introduction of a new serotype or switch in the predominant serotype, which was subsequently followed by outbreaks. Rare cases of autochthonous transmission were reported in countries where most previously reported cases had been imported.

Laboratory sampling schemes and confirmation methods varied by country and area. Some countries in this report were using the 2009 WHO dengue case classification system: (*21*) (i) dengue without warning signs; (ii) dengue with warning signs that include abdominal pain or tenderness, persistent vomiting, clinical fluid accumulation, mucosal bleeding, lethargy or restlessness, liver enlargement and increase in haematocrit with a rapid decrease in platelet count; and (iii) severe dengue, which is characterized by severe plasma leakage, severe haemorrhage and severe organ impairment. Other countries used other case definitions (**Table 1**). Some countries and areas in the Region report all identified cases of dengue, whereas others report only dengue cases at sentinel sites. In addition, some countries and areas conduct active surveillance or vector surveillance, or both (**Table 1**).

### Reporting by country and area

Data for dengue cases were available from 35 countries and areas during this study period, including eight with complete case and death data for all years of this study (**Table 3**). Data were not available for three countries and areas: the Northern Mariana Islands, the Pitcairn Islands and Tokelau.

**Table 3:** Number of dengue cases (including imported cases), number of dengue-attributed deaths and case-fatality ratios reported to WHO from the Western Pacific Region, 2013–2019^a^

Country or area	Year																				
	2013			2014			2015			2016			2017			2018			2019		
	No. of cases	No. of deaths	CFR (%)	No. of cases	No. of deaths	CFR (%)	No. of cases	No. of deaths	CFR (%)	No. of cases	No. of deaths	CFR (%)	No. of cases	No. of deaths	CFR (%)	No. of cases	No. of deaths	CFR (%)	No. of cases	No. of deaths	CFR (%)
**Asia subregion**																					
Brunei Darussalam	2025	–	–	436	2	0.46	–	–	–	–	–	–	–	–	–	–	–	–	–	–	–
Cambodia	17 533	59	0.34	3684	21	0.57	15 412	–	–	12 843	–	–	6372	3	0.05	24 684	23	0.09	68 597	48	0.07
China	4663	0	0.00	46 864	6	0.01	3858	0	0.00	2050	0	0.00	5893	2	0.03	5136	1	0.02	22 188	3	0.01
China, Hong Kong Special Administrative Region	103	0	0.00	112	0	0.00	114	0	0.00	124	–	–	102	–	–	163	–	–	198	–	–
China, Macao Special Administrative Region	9	–	–	17	0	0.00	3	0	0.00	11	–	–	17	–	–	18	–	–	–	–	–
China, Taiwan	596	–	–	15 509	0	0.00	43 467	0	0.00	381	–	–	10	–	–	183	–	–	100	–	–
Japan	249	0	0.00	341	0	0.00	293	0	0.00	338	1	0.30	245	0	0.00	201	0	0.00	463	0	0.00
Lao People's Democratic Republic	44 250	95	0.21	1716	0	0.00	1959	0	0.00	5618	10	0.18	11 067	14	0.13	6446	19	0.29	39 091	76	0.19
Malaysia	43 346	92	0.21	108 698	215	0.20	120 836	336	0.28	109 037	237	0.22	89 487	177	0.20	81 360	147	0.18	130 101	147	0.11
Mongolia	0	0	NA	0	0	NA	0	0	NA	–	–	–	–	–	–	–	–	–	–	–	–
Philippines	204 906	660	0.32	113 485	425	0.37	213 930	647	0.30	220 518	1092	0.50	152 224	811	0.53	216 190	1083	0.50	437 563	1689	0.39
Republic of Korea	251	0	0.00	164	0	0.00	259	0	0.00	313	0	0.00	171	0	0.00	159	0	0.00	273	0	0.00
Singapore	22 170	8	0.04	18 326	6	0.03	11 294	6	0.05	13 085	12	0.09	2767	2	0.07	3283	6	0.18	15 999	3	0.02
Viet Nam	66 322	42	0.06	31 848	30	0.09	97 484	62	0.06	91 609	28	0.03	172 232	40	0.02	131 447	27	0.02	320 702	54	0.02
**Total for subregion**	**406 423**	**956**	**0.24**	**341 200**	**705**	**0.21**	**508 909**	**1051**	**0.21**	**455 927**	**1380**	**0.30**	**440 587**	**1049**	**0.24**	**469 270**	**1306**	**0.28**	**1 035 275**	**2020**	**0.20**
**Pacific subregion**																					
American Samoa	–	–	–	–	–	–	479	4	0.84	0	0	0.00	–	–	–	–	–	–	–	–	–
Australia	1841	0	0.00	1721	0	0.00	1716	0	0.00	2237	0	0.00	1132	1	0.09	917	0	0.00	1463	1	0.07
Cook Islands	–	–	–	946	0	0.00	765	0	0.00	0	–	–	0	–	–	0	–	–	126	0	0.00
Fiji	352	0	0.00	26 595	16	0.06	–	–	–	398	0	0.00	2699	9	0.33	4000	9	0.23	2500	0	0.00
French Polynesia	1523	0	0.00	2155	0	0.00	–	–	–	–	–	–	–	–	–	–	–	–	2400	0	0.00
Guam	–	–	–	–	–	–	–	–	–	–	–	–	–	–	–	–	–	–	23	–	–
Kiribati	–	–	–	–	–	–	–	–	–	0	–	–	0	–	–	1899	2	0.11	–	–	–
Marshall Islands	–	–	–	–	–	–	–	–	–	–	–	–	–	–	–	–	–	–	1635	1	0.06
Federated States of Micronesia (Federated States of)	217	0	0.00	14	0	0.00	1	0	0.00	90	0	0.00	0	–	–	0	–	–	1464	1	0.07
Nauru	–	–	–	251	–	–	–	–	–	0	–	–	964	3	0.31	114	0	0.00	–	–	–
New Caledonia	9958	4	0.04	–	–	–	–	–	–	–	–	–	4200	11	0.26	1997	0	0.00	3916	2	0.05
New Zealand	106	0	0.00	179	0	0.00	125	0	0.00	191	0	0.00	161	0	0.00	294	0	0.00	224	0	0.00
Niue	–	–	–	–	–	–	–	–	–	0	–	–	2	–	–	–	–	–	–	–	–
Northern Mariana Islands	–	–	–	–	–	–	–	–	–	–	–	–	–	–	–	–	–	–	–	–	–
Palau	9	0	0.00	13	2	15.38	20	0	0.00	–	–	–	440	5	1.14	570	2	0.35	737	3	0.41
Papua New Guinea	–	–	–	6	–	–	–	–	–	–	–	–	–	–	–	–	–	–	–	–	–
Pitcairn Islands	–	–	–	–	–	–	–	–	–	–	–	–	–	–	–	–	–	–	–	–	–
Samoa	–	–	–	–	–	–	–	–	–	–	–	–	2724	5	0.18	–	–	–	–	–	–
Solomon Islands	9500	8	0.08	1872	1	0.05	–	–	–	–	–	–	–	–	–	–	–	–	–	–	–
Tokelau	–	–	–	–	–	–	–	–	–	–	–	–	–	–	–	–	–	–	–	–	–
Tonga	–	–	–	51	0	0.00	1559	0	0.00	–	–	–	100	0	0.00	–	–	–	–	–	–
Tuvalu	–	–	–	408	–	–	–	–	–	–	–	–	–	–	–	–	–	–	522	2	0.38
Vanuatu	–	–	–	1561	–	–	–	–	–	–	–	–	> 1 000	–	–	–	–	–	–	–	–
Wallis and Futuna	94	–	–	–	–	–	–	–	–	–	–	–	222	0	0.00	202	–	–	–	–	–
**Total for subregion**	**23 600**	**12**	**0.05**	**35 772**	**19**	**0.05**	**4665**	**4**	**0.09**	**2916**	**0**	**0.00**	**13 644**	**34**	**0.23**	**9993**	**13**	**0.13**	**15 010**	**10**	**0.05**
**TOTAL**	**430 023**	**968**	**0.23**	**376 972**	**724**	**0.19**	**513 574**	**1055**	**0.21**	**458 843**	**1380**	**0.30**	**454 231**	**1083**	**0.24**	**479 263**	**1319**	**0.28**	**1 050 285**	**2030**	**0.19**

CFR: case-fatality ratio; NA: cannot be calculated

^a^ The – symbol indicates that no data were available.

### Asia subregion

#### Brunei Darussalam

Brunei Darussalam reported to WHO 2025 cases in 2013 and 436 cases and 2 deaths (CFR: 0.46%) in 2014. Reports for other years were not available.

#### Cambodia

During 2013–2019, Cambodia annually reported from 6372 to 68 597 suspected cases and from 3 to 59 deaths. The highest number of cases was reported during an outbreak in 2019 that peaked between June and August, with more than 5000 cases reported in epidemiological week 26. (*22*, *23*) The highest number of deaths (59) was reported in 2013 (CFR: 0.34%).

In Cambodia, serotyping was conducted from sentinel laboratory surveillance at five sentinel sites. The predominant serotype reported from 2013 to 2015 was DENV-1, and in 2016, it switched to DENV-2. From the end of 2017 to the end of 2019, the predominant serotype switched back to DENV-1. This latter serotype switch preceded the large-scale outbreak in 2019, during which 73% (details on numerators and denominators are not available) of all serotyped samples between January and July 2019 were DENV-1, and the next most common serotype was DENV-2 (25%), followed by DENV-4 (2.2%) and DENV-3 (0.3%).

#### China

During 2013–2019, China annually reported from 2050 to 46 864 cases (including both clinically and laboratory-confirmed cases) and from 0 to 6 deaths. The highest number of cases and deaths were reported in 2014, with 46 864 cases and 6 deaths (CFR: 0.01%).

Several outbreaks were reported from the southern and central regions of China. Yunnan Province in 2013 reported 1245 cases with 136 that were laboratory-confirmed, no deaths, and a predominant serotype of DENV-3; (*24*) Henan Province in 2013 reported 106 suspected cases, 73 confirmed cases and no deaths, with the predominant serotype being DENV-3; (*25*) Guangdong Province in 2014 accounted for more than 40 000 cases, including 1942 cases that were laboratory-confirmed and hospitalized and 2 deaths, where the predominant serotype among cases was DENV-1. (*6*)

The introduction of a new serotype in China in 2017 caused an outbreak of 1138 autochthonous cases after multiple clades of DENV-2 were introduced to Hangzhou, Zhejiang Province, in a short period. (*26*) During 2013–2019, Hong Kong Special Administrative Region, China, annually reported between 102 and 163 cases. During 2013–2019, China, Taiwan, China, annually reported between 10 and 43 467 cases, with the highest number of cases reported in 2015. During 2013–2018,  Macao Special Administrative Region, China, annually reported between 3 and 18 cases.

#### Japan

During 2013–2019, Japan annually reported between 201 and 463 laboratory-confirmed cases, with 1 death reported in 2016. In 2014, an outbreak of 162 autochthonous dengue cases was reported for the first time in nearly 70 years, of which more than 90% (148/160, from available data) had visited or worked near parks in central Tokyo, and the dominant serotype was DENV-1. (*5*, *27*, *28*)

All cases reported from 2016 to 2018 were imported. The predominant serotype was DENV-2 (36% [61/172] of cases in 2016, 35% [39/113] in 2017, 42% [34/81] in 2018), followed by DENV-3 (23% [39/172] of cases in 2016, 27% [31/113] in 2017, 31% [25/81] in 2018), DENV-1 (31% [54/172] of cases in 2016, 27% [31/113] in 2017, 24% [19/81] in 2018) and DENV-4 (11% [18/172] of cases in 2016, 11% [12/113] in 2017, 4% [3/81] in 2018). In 2019, 17% (78/463) of serotyped cases were DENV-1, 16% (74/463) were DENV-2, 9% (40/463) were DENV-3 and 3% (16/463) were DENV-4.

#### Lao People's Democratic Republic

During 2013–2019, the Lao People's Democratic Republic annually reported between 1716 and 44 250 clinically confirmed cases and 0 to 95 deaths. In 2013, the country reported the largest dengue outbreak in its history, (*14*) with 44 250 cases and 95 deaths reported nationwide. In the southern part of the country alone, 4638 cases and 32 deaths were reported, among which DENV-2, DENV-3 and chikungunya virus were detected, as were concurrent infections with more than one serotype of DENV, or DENV and chikungunya virus. (*29*) More than 90% (numerator not available) of 537 samples serotyped in 2013 were DENV-3. (*30*) In 2015, an outbreak was reported as predominantly due to DENV-1. (*30*) In 2019, there was a dengue outbreak with 39 091 cases reported and 76 deaths (CFR: 0.19%), and 65% (numerator not available) of 1178 specimens collected and serotyped were found to be DENV-2. (*30*) The predominant serotypes during outbreaks in 2013, 2015 and 2019 were attributed to three different serotypes, indicating two serotype switches. (*30*)

#### Malaysia

During 2013–2019, Malaysia annually reported between 43 346 and 130 101 laboratory-confirmed cases and 92 to 336 deaths. No imported cases were reported from 2016 to 2018. Malaysia launched the web-based e-Notification system and e-Dengue system in 2014, and all registered dengue cases since January 2014 have been laboratory-confirmed. More than 100 000 cases were reported in 2014, 2015, 2016 and 2019.

All four serotypes were reported in Malaysia, with the predominant serotype differing each year from 2016 to 2018, with significant cocirculation. In 2016, the predominant serotype was DENV-1 (40%, 2211/5482), followed by DENV-3 (32%, 1745/5482), DENV-2 (25%, 1381/5482) and DENV-4 (3%, 145/5482). In 2017, the predominant serotype was DENV-3 (41%, 2200/5420), followed by DENV-2 (35%, 1887/5420), DENV-1 (23%, 1245/5420) and DENV-4 (2%, 88/5420). In 2018, the predominant serotype was DENV-2 (47%, 2608/5544), followed by DENV-3 (33%, 1833/5544), DENV-1 (19%, 1055/5544) and DENV-4 (1%, 48/5544).

#### Mongolia

During 2013–2015, Mongolia reported no dengue cases and no deaths. Data for 2016–2019 were not available.

#### The Philippines

During 2013–2019, the Philippines annually reported between 113 485 and 437 563 suspected dengue cases and 425 to 1689 deaths. Among these suspected cases, 1488 cases in 2016, 1333 cases in 2017 and 998 cases in 2018 were laboratory-confirmed. The highest number of cases and deaths were reported during a large-scale outbreak in 2019, with 437 563 cases and 1689 deaths (CFR: 0.39%).

All four serotypes were reported from the Philippines. In 2016, the predominant serotype among 1488 cases tested was DENV-1 (44%, 659/1488), followed by DENV-3 (26%, 384/1488), DENV-2 (24%, 349/1488) and DENV-4 (6%, 95/1488); 1 case tested positive for both DENV-1 and DENV-2 (0.1%, 1/1488). In 2017, the predominant serotype among 1333 cases tested was DENV-3 (60%, 795/1333), followed by DENV-1 (24%, 318/1333), DENV-2 (12%, 164/1333) and DENV-4 (4%, 47/1333); 2 cases tested positive for DENV-1 and DENV-2 (0.2%, 2/1333), 5 cases tested positive for DENV-1 and DENV-3 (0.4%, 5/1333) and 2 cases tested positive for DENV-2 and DENV-3 (0.2%, 2/1333). In 2018, the predominant serotype among 998 cases tested was DENV-3 (60%, 598/998), followed by DENV-1 (22%, 223/998), DENV-2 (15%, 149/998) and DENV-4 (3%, 25/988); 2 cases tested positive for DENV-1 and DENV-3 (0.2%, 2/988) and 1 case tested positive for DENV-2 and DENV-3 (0.1%, 1/988). In 2019, the predominant serotype among the 100 cases with serotype data available was DENV-3 (64%), followed by DENV-2 (18%), DENV-1 (15%) and DENV-4 (3%). (*31*)

#### Republic of Korea

During 2013–2019, the Republic of Korea annually reported between 164 and 313 laboratory-confirmed cases and no deaths. The highest number of cases was reported in 2016. All cases reported from 2016 to 2018 were imported, comprising all four serotypes. In 2016, the predominant serotype was DENV-1 (38%, 57/149), followed by DENV-2 (35%, 52/149), DENV-3 (20%, 30/149) and DENV-4 (7%, 10/149). In 2017, the predominant serotype among imported cases was DENV-1 (44%, 38/86), followed by DENV-3 (23%, 20/86), DENV-2 (19%, 16/86) and DENV-4 (14%, 12/86). In 2018, the predominant serotype among imported cases was DENV-2 (37%, 35/96), followed by DENV-1 (33%, 32/96), DENV-3 (28%, 27/96) and DENV-4 (2%, 2/96).

#### Singapore

During 2013–2019, Singapore annually reported between 2767 and 22 170 laboratory-confirmed cases and 2 to 12 deaths. Large numbers of cases were reported during outbreaks in 2013, 2014, 2015 and 2019. The numbers of reported cases were low in 2017 and 2018. Among the 20 deaths reported during 2016–2018, 14 were autochthonous cases and the rest were imported cases. All four serotypes were reported from Singapore; however, denominators were not available, so the percentage for each serotype is reported along with the number of positive cases. The predominant serotypes from 2016 to 2018 were DENV-2 (51% [2257 positive cases] in 2016, 45% [361] in 2017 and 52% [637] in 2018), followed by in 2016 DENV-1 (29%, 278 positive cases) then DENV-3 (18%, 806), and in 2017 DENV-3 (24%, 192) then DENV-1 (21%, 171), and in 2018 DENV-3 (25%, 305) and DENV-1 (20%, 240). DENV-4 was reported from 2% (*n* = 67) of cases in 2016, 10% (77) in 2017 and 4% (47) in 2018.

#### Viet Nam

During 2013–2019, Viet Nam annually reported between 66 322 and 320 702 cases (including both clinically and laboratory-confirmed cases) and 27 to 62 deaths. More than 100 000 cases were reported in 2017, 2018 and 2019; notably, 320 702 cases were reported in 2019. During the outbreak in 2017, more than 59 000 cases were reported in northern Viet Nam, eight times higher than the number of cases in 2016. (*32*)

All four serotypes were reported from Viet Nam during 2016–2018. In 2016, the predominant serotype was DENV-1 (61%, 1104/1803), followed by DENV-4 (25%, 453/1803), DENV-2 (13%, 240/1803) and DENV-3 (0.3%, 6/1803). In 2017, the predominant serotype was DENV-1 (72%, 2057/2870), followed by DENV-2 (21%, 607/2870), DENV-4 (7%, 204/2870) and DENV-3 (0.1%, 2/2870). In 2018, the predominant serotype changed to DENV-2 (50%, 988/1980), followed by DENV-1 (33%, 661/1980), DENV-4 (17%, 328/1980) and DENV-3 (0.2%, 3/1980).

### Pacific subregion

#### Australia

During 2013–2019, Australia annually reported between 917 and 2237 laboratory-confirmed cases and 0 to 1 death. More than 1700 cases were reported annually in 2013, 2014, 2015 and 2016; in 2016, 2237 cases were reported. During 2016–2018, more than 98% of reported cases were imported (2204/2237 in 2016, 1113/1132 in 2017 and 907/917 in 2018). In Australia, dengue cases occur each year in North Queensland, generally originating from an imported case, although in 2019 an outbreak associated with 13 locally acquired cases was reported for the first time in decades in the Rockhampton region, Queensland. (*33*, *34*)

All four serotypes were reported from Australia, with the predominant serotype being DENV-2 (44% [468/1052 of known and serotyped cases] in 2016, 56% [246/436] in 2017, 43% [120/282] in 2018), followed in 2016 by DENV-3 (24%, 257/1052), DENV-1 (19%, 202/1052) and DENV-4 (12%, 125/1052); in 2017 by DENV-1 (20%, 88/436), DENV-3 (13%, 57/436) and DENV-4 (10%, 45/436); and in 2018 by DENV-1 (30%, 86/282), DENV-3 (20%, 55/282) and DENV-4 (7%, 21/282). In addition to these serotyped cases, concurrent infection with two serotypes was reported in 2016 and 2017. In 2016, concurrent infections were reported with DENV-1 and DENV-2 (1 case), DENV-2 and  DENV-3 (1 case), and DENV-3 and DENV-4 (4 cases); in 2017, concurrent infection with DENV-1 and DENV-4 was reported in 1 case; in 2019, concurrent infection with DENV-3 and DENV-4 was reported in 1 case.

#### New Zealand

During 2013–2019, New Zealand annually reported between 106 and 294 cases (including both clinically confirmed and laboratory-confirmed cases, although most are laboratory-confirmed); during 2016–2019, no deaths were reported. Among reported cases, 98% (158/161) were laboratory-confirmed in 2017, 95% (280/294) in 2018 and 98% (219/224) in 2019. (*16*, *35*, *36*) The largest number of cases was reported in 2018, at 294 cases. In 2016, two dengue fever outbreaks were reported that involved 12 cases. During 2013–2019, all cases reported in New Zealand were imported (information on travel history was not available for 1 case in 2015 and 2 cases in 2019).

All four serotypes were reported from New Zealand. In 2016, the predominant serotype was DENV-3 (63%, 59/93), followed by DENV-2 (20%, 19/93), DENV-1 (11%, 10/93) and DENV-4 (5%, 5/93). In 2017 and 2018, the predominant serotype was DENV-2 (83% [82/99] and 84% [167/200], respectively), followed by DENV-1 (10% [10/99] and 9% [18/200], respectively), DENV-3 (6% [6/99] and 5% [9/200], respectively) and DENV-4 (1% [1/99] and 3% [6/200], respectively).

#### American Samoa

American Samoa reported clinically confirmed cases to WHO using the 2009 WHO dengue case classification system. Laboratory confirmation is conducted to confirm outbreaks using reverse transcription polymerase chain reaction (RT–PCR) or an antigen rapid diagnostic test (NS1). In 2015, American Samoa reported 479 cases and 4 deaths (CFR: 0.84%). Outbreaks were also reported in 2017 and 2018, but the total numbers of cases are not available.

#### Cook Islands

The Cook Islands reported clinically confirmed cases to WHO using the 2009 WHO dengue case classification system. In 2014, the Cook Islands reported 946 cases and no deaths, and in 2015 the Islands reported 765 cases and no deaths (CFR: 0%). No cases were reported to WHO during 2016–2018. In 2019, a dengue outbreak was declared in February, with 41 confirmed cases and 85 probable, 48 hospitalizations and no deaths. (*37*) The predominant serotype in 2019 was DENV-1, accounting for 93% (35/38) of cases with available serotype information. Additionally, 3 cases who were tourists with a history of travelling to French Polynesia were confirmed with DENV-2 in October 2019. (*38*)

#### Fiji

During 2013–2018, Fiji annually reported between 352 and 26 595 cases and 0 to 16 deaths. Fiji reported clinically confirmed cases to WHO using the 2009 WHO dengue case classification system. Samples from different health divisions were tested using RT–PCR, an antigen rapid diagnostic test (NS1) and an enzyme-linked immunosorbent assay (ELISA). An outbreak was reported in 2014 of at least 26 595 cases (more than 27 000 reported according to some sources) and 16 deaths (CFR: 0.06%). From 2017 to 2018, an outbreak was reported, for which the predominant serotype was DENV-2. (*39*)

#### French Polynesia

French Polynesia used the 2009 WHO dengue case classification system, and the laboratory method for confirmation was RT–PCR. In 2013, French Polynesia reported 1523 dengue cases associated with an outbreak, with 258 being laboratory-confirmed; during the outbreak, 70% (170/244) of cases with the serotype identified had DENV-1 infections, 30% (73/244) had DENV-3 infections (genotype III) and 0.4% (1/244) had coinfection with both serotypes. (*40*) DENV-3 was reported to have been introduced from South America. (*40*) In 2014, 2155 confirmed and 34 000 suspected cases were reported in French Polynesia, and outbreaks were also reported in 2016 and 2017. In 2016 and 2017, DENV-1 was reported, and in 2018, DENV-2 was reported. In April 2019, an outbreak of DENV-2 was declared, with 2400 autochthonous cases reported. (*41*)

#### Guam

Guam reported clinically confirmed cases to WHO: 23 cases were reported in 2019, with no further information available.

#### Kiribati

Kiribati reported clinically confirmed cases to WHO using the 2009 WHO dengue case classification system. Laboratory testing to confirm outbreaks is conducted using RT–PCR or an antigen rapid diagnostic test (NS1). In Kiribati, outbreaks were reported in 2013 and 2014, and no cases were reported in 2016 and 2017. In 2018, 1899 cases and 2 deaths were reported, with DENV-2 detected.

#### Republic of the Marshall Islands

In the Republic of the Marshall Islands, outbreaks were reported in 2013 and 2014. In 2019, a DENV-3 outbreak was reported with at least 1395 cases of dengue-like illness, including 431 laboratory-confirmed cases and 1 death. (*42*) A health emergency was declared in relation to this event; internal movement restrictions were imposed between the affected and unaffected islands; and emergency medical teams were deployed to support the dengue response.

#### Federated States of Micronesia

The Federated States of Micronesia reported clinically confirmed cases to WHO using the 2009 WHO dengue case classification system. Laboratory methods used to confirm outbreaks include RT–PCR and an antigen rapid diagnostic test (NS1). There were 217 cases reported to WHO in 2013, associated with an outbreak of 729 suspected dengue cases and no deaths in Kosrae from September 2012 to March 2013. DENV-4 was detected from 3 specimens collected during this period; 11% (728/6600) of Kosrae residents met the case definition for suspected dengue, and almost 4% (242/6600) were hospitalized. (*43*) In 2018, DENV-4 was reported. In 2019, 1464 dengue cases including 1 death were reported from Yap state, and the predominant serotype was DENV-3. The dengue outbreak in 2019 coincided with a concurrent leptospirosis outbreak in Yap state, and an executive order determining a public health crisis was issued.

#### Nauru

Nauru reported clinically confirmed cases to WHO using the 2009 WHO dengue case classification system. Laboratory testing to confirm outbreaks uses RT–PCR or an antigen rapid diagnostic test (NS1). Nauru reported 251 cases in 2014, no cases in 2016, 964 cases and 3 deaths in 2017 and 114 cases and no deaths in 2018. In 2017, DENV-2 was reported and in 2018, DENV-1 was reported.

#### New Caledonia

New Caledonia reported cases to WHO using the 2009 WHO dengue case classification system and RT–PCR for laboratory confirmation. In 2013, New Caledonia reported 9958 cases including 4 deaths during an outbreak in which the predominant serotype was DENV-1. (*44*) Based on available information, an outbreak was also reported in 2014. In 2017, 4200 cases and 11 deaths were reported, with DENV-1, DENV-2 and DENV-3 detected. From November 2018 to September 2019, a dengue outbreak was declared. From 1 January to 31 December 2019, 3916 cases, 368 hospitalizations and 2 deaths were reported. Among the 316 cases with serotype information available, the predominant serotype was DENV-2. Two cases of DENV-1 and 1 case of DENV-4 were imported from French Polynesia and Indonesia, respectively. (*45*)

#### Niue

Niue reported clinically confirmed cases to WHO. In Niue, 2 cases were reported in 2017. In 2018, DENV-2 was reported, but information on the number of cases was not available.

#### Palau

Palau reported cases to WHO using the 2009 WHO dengue case classification system and RT–PCR or an antigen rapid diagnostic test (NS1) for laboratory testing to confirm outbreaks. During 2013–2017, Palau annually reported between 9 and 737 cases and 0 to 5 deaths. Outbreaks were reported in 2016 and again in 2017, the latter comprising 440 cases and 5 deaths, with a predominant serotype of DENV-2. In 2018, 570 cases and 2 deaths were reported, and in 2019, there were 737 cases including 3 deaths. From December 2018 to September 2019, 160 cases were confirmed as DENV-3. Two serotypes were reported from Palau; DENV-2 was reported in 2016 and 2017, and DENV-3 was reported in 2018.

#### Papua New Guinea

In 2014, Papua New Guinea reported 6 cases. Further information was not available.

#### Samoa

Samoa reported cases to WHO using the 2009 WHO dengue case classification system and RT–PCR or an antigen rapid diagnostic test (NS1) to confirm outbreaks. In Samoa, outbreaks were reported in 2015 and 2016. In 2017, 2724 cases and 5 deaths were reported, with the predominant serotype being DENV-3. In 2018, DENV-2 was reported.

#### Solomon Islands

The Solomon Islands reported cases to WHO using the 2009 WHO dengue case classification system. In the Solomon Islands in 2013, 9500 cases and 8 deaths (CFR: 0.10%) were associated with an outbreak in Honiara. DENV-3 genotype I was isolated from specimens collected during this outbreak, suggesting introduction from south-eastern Asia after 18 years of dengue absence in the PICs. (*40*) In 2014, 1872 cases and 1 death (CFR: 0.05%) were reported. The introduction of DENV-2 to the Solomon Islands resulted in outbreaks in 2016 and 2017. (*46*, *47*) From September 2016 to April 2017, an outbreak of DENV-2 was reported in 9 of 10 provinces in the Solomon Islands, with 12 329 suspected cases, including 1510 cases positive by dengue rapid diagnostic test, and 16 deaths. (*47*) An outbreak was also reported in 2019.

#### Tonga

Tonga reported cases to WHO using the 2009 WHO dengue case classification system. In Tonga, 51 cases and no deaths were reported in 2014; 1559 cases and no deaths were reported in 2015; and more than 100 cases were reported in 2017.

#### Tuvalu

Tuvalu reported cases to WHO using the 2009 WHO dengue case classification system. In Tuvalu, 408 cases were reported in 2014. In March 2019, a dengue outbreak was declared. In 2019, 522 cases were reported, including at least 21 hospitalizations and 2 deaths in children. The predominant serotype in the 2019 outbreak was DENV-1.

#### Vanuatu

Vanuatu reported clinically confirmed cases to WHO. In Vanuatu, 1561 cases were reported in 2014 and more than 1000 cases were reported in 2017; DENV-2 was reported in 2018.

#### Wallis and Futuna

Wallis and Futuna reported cases to WHO using the 2009 WHO dengue case classification system. In Wallis and Futuna, 94 cases were reported in 2013. In 2017, an outbreak was declared in November, with 222 cases and no deaths, and DENV-1 was identified from 2 samples. In 2018, 202 cases and DENV-1 were reported. In November 2019, an outbreak was declared in Wallis and Futuna, and 30 confirmed cases were reported from February to December 2019, with the predominant serotype being DENV-2. (*48*)

## Discussion

Dengue continued to pose a health burden in the Region during 2013–2019, with the number of annually reported cases ranging from a little more than 430 000 to more than 1 million and with the annual number of reported deaths ranging from 724 to 2025. Outbreaks were reported from the Region every year during the study period. The introduction or reintroduction of serotypes to specific areas caused several outbreaks and rare occurrences of local transmission in places where dengue had not been previously reported. With support from countries and areas, WHO continued to share timely information during the study period through its biweekly dengue epidemiological reports for the Region (*18*) and conducted regional and country-specific risk assessments to inform dengue prevention and control efforts.

The increases in reported cases and regional case incidence may be attributed to several factors. First, a true increase in dengue incidence may have occurred due to expanding urbanization and increasing population size and density, particularly in settings with increased exposure to competent dengue vectors and mosquito breeding grounds. (*49*) Shifts in ecological factors due to climate change, such as intensified rainy seasons and higher ambient temperatures, have expanded the geographical range of *Aedes* mosquitos globally during the past 50 years and led to intensified dengue transmission. (*50*) Second, increased international travel and trade have led to the importation of cases with different serotypes and the introduction of mosquito eggs through the importation of goods to areas where the population is susceptible and competent mosquitos exist. (*49*, *50*) Third, reports to national health authorities likely increased due to strengthened surveillance systems and diagnostic capacities, including laboratory networks that supported confirmatory diagnosis in the PICs, as well as an emphasis on risk communication activities to improve the awareness of dengue among the public. (*3*) The range of CFRs may be associated with differences in case reporting, the timing of the case presentation to health-care facilities and clinical management.

The number of cases reported in 2019 was higher than in the years from 2013 to 2018, and the CFR was relatively low. This increase in 2019 included at least 14 countries and areas that reported dengue outbreaks in the Region, including large-scale outbreaks; during 2019, four countries and areas in the Asia subregion and three in the Pacific subregion reported their highest number of cases of the 7-year study. It is possible that case detection and reporting increased due to improved awareness of dengue among health-care professionals and the public because of the large outbreaks. These outbreaks may have also increased health-care-seeking behaviour, leading to fewer deaths, thereby decreasing the CFR.

Our findings show that there is a substantial burden of dengue in the Region and that it continues to increase over time. However, dengue surveillance practices throughout the Region are inconsistent and require strengthening. To inform national and regional risk assessments and actions, information is required not only on the time, place and demographics of a case, such as age and sex, but also on the DENV serotype and whether the infection was locally acquired or imported. These details will also support risk assessments for and responses to events with new epidemiological patterns, such as outbreaks associated with the introduction or reintroduction of serotypes to specific areas, as well as rare occurrences of local transmission in places where it was not previously reported. Furthermore, in some settings, the capacities for surveillance, outbreak response, clinical management and diagnosis may be limited. Several approaches could fill these gaps, including strengthening laboratory capacity and laboratory networks, institutionalizing active surveillance to detect dengue cases who are self-managed and inapparent, and implementing integrated vector surveillance.

Although several countries and areas have adopted the 2009 WHO dengue case classification system, (*21*) there are differences in countries and areas across the Region in surveillance methodology, including whether universal or sentinel reporting is used; laboratory sampling schemes and confirmation methods; and reporting practices. These differences are a limitation of this report, indicating why comparison across countries should be avoided and comparisons within one country should be informed by the local reporting practices, which may change over time. As a result of differences in case definitions and other factors, there is likely to be underreporting and, thus, an underestimation of the true regional burden in terms of the number of cases, CFRs and incidence. (*1*, *2*) Despite these limitations, continued reporting of dengue in line with the Regional Action Plan is important to guide public health authorities in their national and subnational response efforts.

The burden of dengue, including the increased risks of dengue outbreaks, will continue amid other public health emergencies. Disaggregating data by age and sex at all levels will enable public health authorities to implement improved and targeted response measures. Additional information about cases, including their travel history and serotype, should also be routinely collected and reported. The Region’s capacity to mitigate the impact of dengue can be strengthened by making a shift in its management, from a reactive, acute outbreak response to one that reduces fatalities through undertaking activities, including sustainable implementation of mosquito control measures, engaging communities to raise their awareness about the risk of dengue and to communicate relevant behavioural changes, and strengthening diagnostics and case management. Enhancing collaboration and coordination within and beyond the health sector is key to carrying out these activities successfully.

## References

[1] Dengue and severe dengue: key facts. Geneva: World Health Organization; 2022. Available from: https://www.who.int/news-room/fact-sheets/detail/dengue-and-severe-dengue, accessed 17 June 2022.

[2] Bhatt S, Gething PW, Brady OJ, Messina JP, Farlow AW, Moyes CL, et al. The global distribution and burden of dengue. Nature. 2013 Apr 25;496(7446):504–7. 10.1038/nature12060 23563266 PMC3651993

[3] Western Pacific regional action plan for dengue prevention and control (2016). Manila: WHO Regional Office for the Western Pacific; 2017. Available from: https://apps.who.int/iris/handle/10665/258651, accessed 17 June 2022.

[4] Lao M, Caro V, Thiberge JM, Bounmany P, Vongpayloth K, Buchy P, et al. Co-circulation of dengue virus type 3 genotypes in Vientiane capital, Lao PDR. PLoS One. 2014 12 31;9(12):e115569. 10.1371/journal.pone.0115569 25551768 PMC4281081

[5] Arima Y, Matsui T, Shimada T, Ishikane M, Kawabata K, Sunagawa T, et al. Ongoing local transmission of dengue in Japan, August to September 2014. West Pac Surveill Response. 2014 10 28;5(4):27–9. 10.5365/wpsar.2014.5.3.007 25685602 PMC4318974

[6] Zhao H, Zhang FC, Zhu Q, Wang J, Hong WX, Zhao LZ, et al. Epidemiological and virological characterizations of the 2014 dengue outbreak in Guangzhou, China. PLoS One. 2016 06 3;11(6):e0156548. 10.1371/journal.pone.0156548 27257804 PMC4892648

[7] Aguas R, Dorigatti I, Coudeville L, Luxemburger C, Ferguson NM. Cross-serotype interactions and disease outcome prediction of dengue infections in Vietnam. Sci Rep. 2019 06 28;9(1):9395. 10.1038/s41598-019-45816-6 31253823 PMC6598999

[8] Arima Y, Chiew M, Matsui T; Emerging Disease Surveillance and Response Team, Division of Health Security and Emergencies, World Health Organization Regional Office for the Western Pacific. Epidemiological update on the dengue situation in the Western Pacific Region, 2012. West Pac Surveill Response. 2015 04 20;6(2):82–9. 10.5365/wpsar.2014.5.4.002 26306221 PMC4542491

[9] Arima Y, Edelstein ZR, Han HK, Matsui T. Epidemiologic update on the dengue situation in the Western Pacific Region, 2011. West Pac Surveill Response. 2013 05 14;4(2):47–54. 10.5365/wpsar.2012.3.4.019 24015372 PMC3762964

[10] Arima Y, Matsui T. Epidemiologic update of dengue in the Western Pacific Region, 2010. West Pac Surveill Response. 2011 06 27;2(2):4–8. 10.5365/wpsar.2011.2.2.005 23908882 PMC3730957

[11] Dengue virus infection – surveillance case definition. Canberra: Australian Government Department of Health and Aged Care; 2018. Available from: https://www.health.gov.au/resources/publications/dengue-virus-infection-surveillance-case-definition, accessed 17 June 2022.

[12] 2018 national guideline for clinical management of dengue. Phnom Penh: Cambodia Ministry of Health; 2018. Available from: https://niph.org.kh/niph/uploads/library/pdf/GL055_National_guideline_for_ClM_Dengue.pdf, accessed 17 June 2022.

[13] Dengue fever Tokyo: Ministry of Health, Labour and Welfare; 2019. Available from: https://www.mhlw.go.jp/bunya/kenkou/kekkaku-kansenshou11/01-04-19.html, accessed 17 June 2022 (in Japanese).

[14] Khampapongpane B, Lewis HC, Ketmayoon P, Phonekeo D, Somoulay V, Khamsing A, et al. National dengue surveillance in the Lao People’s Democratic Republic, 2006-2012: epidemiological and laboratory findings. Western Pac Surveill Response J. 2014 03 31;5(1):7–13. 10.5365/wpsar.2014.5.1.001 24734212 PMC3984965

[15] Case definitions for infectious diseases in Malaysia, third edition. Putrajaya: Ministry of Health; 2017. Available from: https://www2.moh.gov.my/moh/resources/Penerbitan/Garis%20Panduan/Pengurusan%20KEsihatan%20&%20kawalan%20pykit/Case_Definition_Of_Infectious_Disease_3rd_Edition_2017.pdf, accessed 17 June 2022.

[16] Institute of Environmental Science and Research. Notifiable diseases in New Zealand: annual report 2019. Porirua (New Zealand): Institute of Environmental Science and Research; 2021. Available from: https://surv.esr.cri.nz/PDF_surveillance/AnnualRpt/AnnualSurv/2019/2019AnnualNDReport_FINAL.pdf, accessed 28 October 2022.

[17] Arboviral diseases: part of the Communicable Disease Control Manual. Wellington: Ministry of Health New Zealand; 2012. Available from: https://www.health.govt.nz/our-work/diseases-and-conditions/communicable-disease-control-manual/arboviral-diseases, accessed 17 June 2022.

[18] Surveillance – dengue. Manila: WHO Regional Office for the Western Pacific; 2022. Available from: https://www.who.int/westernpacific/emergencies/surveillance/dengue, accessed 17 June 2022.

[19] World population prospects 2022. New York: United Nations Department of Economic and Social Affairs; 2022. Available from: https://population.un.org/wpp/, accessed 30 October 2022.

[20] Disease outbreaks. Cairo: WHO Regional Office for the Eastern Mediterranean; 2022. Available from: http://www.emro.who.int/health-topics/disease-outbreaks/index.html, accessed 15 July 2022.

[21] Dengue guidelines for diagnosis, treatment, prevention and control: new edition. Geneva: World Health Organization; 2009. Available from: https://apps.who.int/iris/handle/10665/44188, accessed 17 June 2022. 23762963

[22] Dengue increase likely during rainy season: WHO warns. Manila: WHO Regional Office for the Western Pacific; 2019. Available from: https://www.who.int/westernpacific/news/detail/11-06-2019-dengue-increase-likely-during-rainy-season-who-warns, accessed 17 June 2022.

[23] Dengue situation update number 585. Manila: WHO Regional Office for the Western Pacific; 2020. Available from: https://apps.who.int/iris/bitstream/handle/10665/330698/Dengue-20200102.pdf?sequence=1, accessed 17 June 2022.

[24] Zhang FC, Zhao H, Li LH, Jiang T, Hong WX, Wang J, et al. Severe dengue outbreak in Yunnan, China, 2013. Int J Infect Dis. 2014 Oct;27:4–6. 10.1016/j.ijid.2014.03.1392 25107464

[25] Huang XY, Ma HX, Wang HF, Du YH, Su J, Li XL, et al. Outbreak of dengue Fever in central China, 2013. Biomed Environ Sci. 2014 Nov;27(11):894–7. 10.3967/bes2014.125 25374022

[26] Yu H, Kong Q, Wang J, Qiu X, Wen Y, Yu X, et al. Multiple lineages of dengue virus serotype 2 cosmopolitan genotype caused a local dengue outbreak in Hangzhou, Zhejiang province, China, in 2017. Sci Rep. 2019 05 14;9(1):7345. 10.1038/s41598-019-43560-5 31089152 PMC6517437

[27] Kutsuna S, Kato Y, Moi ML, Kotaki A, Ota M, Shinohara K, et al. Autochthonous dengue fever, Tokyo, Japan, 2014. Emerg Infect Dis. 2015 Mar;21(3):517–20. 10.3201/eid2103.141662 25695200 PMC4344289

[28] Cases of domestic infection with dengue fever: 38th report Tokyo: Japan Ministry of Health, Labour and Welfare; 2014. Available from: https://www.mhlw.go.jp/bunya/kenkou/kekkaku-kansenshou19/dl/20141031-01.pdf, accessed 17 June 2022 (in Japanese).

[29] Phommanivong V, Kanda S, Shimono T, Lamaningao P, Darcy AW, Mishima N, et al. Co-circulation of the dengue with chikungunya virus during the 2013 outbreak in the southern part of Lao PDR. Trop Med Health. 2016 08 4;44(1):24. 10.1186/s41182-016-0020-y 27524929 PMC4973078

[30] Calvez E, Pommelet V, Somlor S, Pompon J, Viengphouthong S, Bounmany P, et al. Trends of the dengue serotype-4 circulation with epidemiological, phylogenetic, and entomological insights in Lao PDR between 2015 and 2019. Pathogens. 2020 09 3;9(9):728. 10.3390/pathogens9090728 32899416 PMC7557816

[31] Epidemiology Bureau, Public Health Surveillance. Philippine integrated disease surveillance and response (PIDSR): morbidity week no. 52. Manila: Philippines Department of Health, Epidemiology Bureau; 2019. Available from: https://doh.gov.ph/sites/default/files/statistics/PIDSR%20Weekly%20Surveillance%20Report%20No.%2052%202019.pdf, accessed 17 June 2022.

[32] Nguyen HV, Than PQT, Nguyen TH, Vu GT, Hoang CL, Tran TT, et al. Knowledge, attitude and practice about dengue fever among patients experiencing the 2017 outbreak in Vietnam. Int J Environ Res Public Health. 2019 03 18;16(6):976. 10.3390/ijerph16060976 30889912 PMC6466316

[33] Dengue: virus, fever and mosquitos. Brisbane: Queensland Health; 2021. Available from: https://www.health.qld.gov.au/clinical-practice/guidelines-procedures/diseases-infection/diseases/mosquito-borne/dengue/virus-fever, accessed 28 October 2022.

[34] Walker J, Pyke A, Florian P, Moore F, Smoll N, Adegbija O, et al. Re-emergence of dengue virus in regional Queensland: 2019 dengue virus outbreak in Rockhampton, Central Queensland, Australia. Commun Dis Intell. 2018;45:10.33321/cdi.2021.45.31. doi:10.33321/cdi.2021.45.31 10.33321/cdi.2021.45.31 34139967

[35] Institute of Environmental Science and Research. Notifiable diseases in New Zealand: annual report 2017. Porirua (New Zealand): Institute of Environmental Science and Research; 2019. Available from: https://surv.esr.cri.nz/PDF_surveillance/AnnualRpt/AnnualSurv/2017/2017AnnualNDReport_FINAL.pdf, accessed 28 October 2022.

[36] Institute of Environmental Science and Research. Notifiable diseases in New Zealand: annual report 2018. Porirua (New Zealand): Institute of Environmental Science and Research; 2020. Available from: https://surv.esr.cri.nz/PDF_surveillance/AnnualRpt/AnnualSurv/2018/2018AnnualNDReport_FINAL.pdf, accessed 28 October 2022.

[37] Dengue-1 outbreak in the Cook Islands: situation report 8: ending 31 December 2019. Reporting date: 15 January 2020. Rarotonga: Cook Islands Ministry of Health; 2020. Available from: https://reliefweb.int/report/cook-islands/dengue-1-outbreak-cook-islands-situation-report-8-ending-31-december-2019, accessed 17 June 2022.

[38] Dengue-1 outbreak in the Cook Islands: situation report 7: ending 30 November 2019. Reporting date: 06 December 2019. Rarotonga: Cook Islands Ministry of Health; 2019. Available from: https://reliefweb.int/report/cook-islands/dengue-1-outbreak-cook-islands-situation-report-7-ending-30-november-2019, accessed 17 June 2022.

[39] Dengue fever [website]. Suva: Fiji Ministry of Health and Medical Services; 2020. Available from: https://www.health.gov.fj/dengue-fever/, accessed 2 October 2020.

[40] Cao-Lormeau VM, Roche C, Musso D, Mallet HP, Dalipanda T, Dofai A, et al. Dengue virus type 3, South Pacific Islands, 2013. Emerg Infect Dis. 2014 Jun;20(6):1034–6. 10.3201/eid2006.131413 24856252 PMC4036764

[41] Dengue situation update number 584. Manila: WHO Regional Office for the Western Pacific; 2019. Available from: https://apps.who.int/iris/bitstream/handle/10665/279856/Dengue-20191219.pdf?sequence=25&isAllowed=y, accessed 17 June 2022.

[42] Dengue-3 outbreak in Republic of the Marshall Islands, June 25–December 1 2019. Situation report date: December 1, 2019. Majuro: Government of the Marshall Islands; 2019. Available from: https://reliefweb.int/report/marshall-islands/dengue-3-outbreak-republic-marshall-islands-june-25-december-1-situation, accessed 17 June 2022.

[43] Centers for Disease Control and Prevention (CDC). Dengue outbreak–Federated States of Micronesia, 2012-2013. MMWR Morb Mortal Wkly Rep. 2013 Jul 19;62(28):570–3. 23863704 PMC4604812

[44] Dupont-Rouzeyrol M, Aubry M, O’Connor O, Roche C, Gourinat AC, Guigon A, et al. Epidemiological and molecular features of dengue virus type-1 in New Caledonia, South Pacific, 2001-2013. Virol J. 2014 03 31;11(1):61. 10.1186/1743-422X-11-61 24684835 PMC3997821

[45] La dengue, le chikungunya et le Zika [Dengue, chikungunya and Zika]. Nouméa: Government of New Caledonia, Directorate of Health and Social Affairs; 2020. Available from: https://dass.gouv.nc/votre-sante-maladies/la-dengue-le-chikungunya-et-le-zika, accessed 2 October 2020 (in French).

[46] Darcy AW, Kanda S, Dalipanda T, Joshua C, Shimono T, Lamaningao P, et al. Multiple arboviral infections during a DENV-2 outbreak in Solomon Islands. Trop Med Health. 2020 05 15;48(1):33. 10.1186/s41182-020-00217-8 32435149 PMC7225641

[47] Craig AT, Joshua CA, Sio AR, Teobasi B, Dofai A, Dalipanda T, et al. Enhanced surveillance during a public health emergency in a resource-limited setting: Experience from a large dengue outbreak in Solomon Islands, 2016-17. PLoS One. 2018 06 7;13(6):e0198487. 10.1371/journal.pone.0198487 29879179 PMC5991673

[48] Agence de santé du territoire des îles Wallis et Futuna: Bulletin de surveillance épidémiologique 02/2020 (semaines 3–4) [Health Agency of the Islands of Wallis and Futuna: Epidemiological surveillance bulletin 02/2020 (weeks 3–4)]. Mata Utu: Wallis and Futuna; 2020. Available from: https://reliefweb.int/report/wallis-and-futuna-france/agence-de-sant-du-territoire-des-les-wallis-et-futuna-bulletin-de-20, accessed 17 June 2022 (in French).

[49] Gubler DJ. Dengue, urbanization and globalization: the unholy trinity of the 21st century. Trop Med Health. 2011 Dec;39(4) Suppl:3–11. 10.2149/tmh.2011-S05 22500131 PMC3317603

[50] Ebi KL, Nealon J. Dengue in a changing climate. Environ Res. 2016 Nov;151:115–23. 10.1016/j.envres.2016.07.026 27475051

[51] Guidelines for the national implementation of dengue rapid diagnostic test (RDT). Manila: Philippines Department of Health; 2016. Available from: https://www.scribd.com/document/459819595/Guidelines-for-the-National-Implementation-of-Dengue-Rapid-Diagnostic-Test-RDT, accessed 18 July 2022.

[52] Revised dengue clinical case management guidelines 2011. Manila: Philippines Department of Health; 2012. Available from: https://psbimannualreview.weebly.com/uploads/4/5/4/5/45454099/revised_dengue_clinical_case_management_guidelines_2011-doh.pdf, accessed 18 July 2022.

[53] Dengue. Manila: Philippines Department of Health; 2020. Available from: https://doh.gov.ph/Health-Advisory/Dengue, accessed 17 June 2022.

[54] 2019 viral mosquito-borne disease management guidelines Cheongju: Korea Disease Control and Prevention Agency; 2019. Available from: http://www.cdc.go.kr/board.es?mid=a20507020000&bid=0019&act=view&list_no=143975, accessed 17 June 2022 (in Korean).

[55] Rajarethinam J, Ang LW, Ong J, Ycasas J, Hapuarachchi HC, Yap G, et al. Dengue in Singapore from 2004 to 2016: cyclical epidemic patterns dominated by serotypes 1 and 2. Am J Trop Med Hyg. 2018 Jul;99(1):204–10. 10.4269/ajtmh.17-0819 29848407 PMC6085773

[56] Circular No. 54/2015/TT-BYT: Guidelines and report information to declare the disease, infectious disease Hanoi: Viet Nam Ministry of Health; 2015. Available from: http://vbpl.yte.gov.vn/van-ban-phap-luat/thong-tu-542015tt-byt.6.1508.html, accessed 17 June 2022 (in Vietnamese).

